# Knowledge, Attitudes, and Beliefs of Medical Students Toward Transgender Healthcare: A Community-Driven Initiative

**DOI:** 10.7759/cureus.49992

**Published:** 2023-12-05

**Authors:** Tonoya Sengupta, Tripti Soni, Alexa M Bolock, Sarah A Heisey, Elizabeth C Kuchinski, Brian J Piper, Jennifer M Joyce, Christian J Carbe

**Affiliations:** 1 Department of Medical Education, St. George's University School of Medicine, St. George's, GRD; 2 Department of Psychiatry, University of Maryland Medical Center, Baltimore, USA; 3 Department of Radiology, New York-Presbyterian Weill Cornell Medical College, New York, USA; 4 Department of Medicine, Prisma Health Greenville Memorial Hospital, Greenville, USA; 5 Department of Medical Education, Geisinger Commonwealth School of Medicine, Scranton, USA

**Keywords:** lgbtq medicine, culturally competent care, transgender and gender non-conforming, transgender community, transgender care, community healthcare, gender-diverse, undergraduate medical education, gender-affirming care, transgender health

## Abstract

Introduction

Transgender patients face substantial systemic healthcare barriers and inadequate care from providers who often demonstrate clinical gaps in the medical needs of the transgender community. Providing interventions in which affirming transgender healthcare is explored, is crucial to delivering competent transgender-patient care and building compassionate physician-patient relationships. The Northeast Pennsylvania (NEPA) Trans Health Conference was established to address the growing need for an educational forum where transgender people could voice their narratives. In this educational intervention study, changes in the knowledge, attitudes, and beliefs about the psychosocial and medical needs of the transgender community in first-year undergraduate medical students were examined pre- and post-trans health conference attendance.

Materials and methods

In the late spring of both 2018 and 2019, first-year medical students attended the NEPA Trans Health Conference, hosted by the Geisinger Commonwealth School of Medicine (GCSOM). Student knowledge, attitudes, and beliefs, regarding the healthcare needs of the transgender community were evaluated prior to and directly after the conference (intervention). Though the surveys shared thematic similarities, the 2018 and 2019 surveys were different and thus were not used comparatively.

Results

In 2018, 35.24% of first-year medical students (37/105 participants) completed both the pre- and post-survey. Overall, 62.5% (5/8) of survey items yielded significant differences. In 2019, 25.5%, of first-year medical students (28/110 participants) completed both the pre- and post-survey and 47.6% (9/21) of survey items yielded significant results. Overall, although the majority of first-year medical students displayed positive attitudes toward trans people pre-intervention, the students also demonstrated increased knowledge, empathy, and understanding of the transgender healthcare narrative post-intervention.

Conclusion

Providing medical students with a humanistic intervention within the medical curriculum that is focused on the transgender person, in addition to their past and present healthcare experiences, offers a bridge between academic content and providing inclusive gender-affirming healthcare to all patients.

## Introduction

Transgender patients experience pronounced healthcare disparities compared to their cisgender counterparts, across age and sexual orientation [1,2]. Disparities in the treatment of transgender patients due to deficiencies in cultural competency perpetuate poor health outcomes, such as suicide, substance misuse, depression, harassment, victimization, and HIV/AIDS [3-6]. Individuals within the transgender community often face systemic barriers within the medical field, such as lack of comprehensive access to health insurance, discrimination from providers, and incompetent provider training in transgender-specific health needs [7,8]. The omission of LGBTQIA2S+-specific education and training dedicated during medical school and residency results in inadequate student awareness and exposure, furthering these disparities [9-11]. Multidisciplinary medical needs of transgender patients are often inadequately met due to a lack of competent training by the healthcare teams, and thus, addressing these insufficiencies can lead to significantly positive outcomes in short and long-term physical and socioemotional well-being [1,12-15].

Transgender, gender non-binary, and genderqueer patients with exposure to clinicians with lower perceived levels of knowledge regarding patient-specific care knowledge have been associated with significantly higher odds of lower self-rated health status and severe psychological distress [16]. Physicians and medical students report having a lack of exposure, inadequate formal education, and substantial clinical training gaps in their knowledge of transgender healthcare [17-20]. Providing structured curricula regarding transgender health holistically benefits student knowledge and is crucial to building trusting relationships between healthcare provider and patient, so that patients feel comfortable in honestly sharing personal information regarding health, sexual orientation, and gender identity [21-24]. Medical schools that provide students with information and skills regarding competent transgender healthcare also need to examine the intersection of disparities that impact wellness, like geography, religion, language and literacy, culture, economic class, race, and age [6,21].

Since 2012, Geisinger Commonwealth School of Medicine (GCSOM) has hosted the Northeast Pennsylvania (NEPA) Trans Health Conference. Originally proposed by a medical student attending GCSOM, the NEPA Trans Health Conference was conceived as an educational forum for NEPA health care professionals and their staff, educators, and other community members to learn with and from the transgender community. Respected physical and behavioral health clinicians who treat and support the LGBTQIA2S+ community share their expertise about the respect, compassion, and current clinical guidelines, available to improve the care for those who identify as transgender [25,26]. An underlying goal for this conference, since its inception, has been to reduce provider knowledge gaps and avoid the potential psychological harm and medical neglect incurred by transgender people due to inadequate transgender healthcare [26]. 

In this study, we examined the changes in knowledge, attitudes, and beliefs of the psychosocial and medical needs of the transgender community in first-year undergraduate medical students attending the NEPA Trans Health Conference and discussed the importance of longitudinal interventions to improve skills and sensitivity of future physicians to provide compassionate and competent gender diverse and transgender healthcare.

## Materials and methods

Study setting

A pre-post interventional study design was employed, with first-year undergraduate medical students a month out from completing their first full academic year, who attended the Northeast Pennsylvania (NEPA) Trans Health Conference at Geisinger Commonwealth School of Medicine in 2018 and 2019. Students were surveyed on their knowledge, attitudes, and beliefs about the psychosocial and medical needs of the transgender community prior to and subsequently following the conference intervention. The annual NEPA Trans Health Conference took place in Scranton, Pennsylvania, at GCSOM in 2018 and 2019. This conference is organized entirely by volunteers from the transgender community and their families; medical students and graduate students from GCSOM, behavioral health professionals, physicians, and educators. The NEPA Trans Health Conference serves to establish a campus and community partnership for improving health care by learning from and with the communities served how best to meet their needs [26]. This study was approved by the Wright Center for Graduate Medical Education’s Institutional Review Board under the project title: Awareness of beliefs, attitudes, and basic medical knowledge among 1st and 2nd year medical students regarding patients that identify as transgender in the clinic, (approval number: 1366009-2).

Participants

2018 and 2019 conference presenters and speakers included relatives of gender nonconforming family members, elementary school guidance counselors, and principals; educators and trans activists from pre-school through college; social workers, therapists, physicians, and lawyers specializing in trans care; as well as local government officials as well as the Acting Secretary and Physician General for the Commonwealth of Pennsylvania. The conference was open to all community members, medical professionals, students, families, and advocates, and staff at GCSOM with a notable inclusion and appreciation for the trans and nonbinary people and their families and loved ones who attended, presented, and participated in the conference events. Study participants included all first-year medical students attending GCSOM. Before the conference, students were informed about the purpose and aim of the study, namely, to investigate the attitudes, beliefs, and level of knowledge of the psychosocial and medical needs of the transgender community in medical students via a pre- and post-conference survey. Additional instructions regarding survey participation and data security were explained to the students via class announcements by peers and conference organizers. Though conference attendance was mandatory for all first-year students, students were explicitly told that survey participation was voluntary and had no impact on class standings or grades.

Procedures

For each single-day conference in 2018 and 2019, all first-year medical students were required to attend three hours of didactic breakout sessions of their choosing from the scheduled eight hours of activities and training. The 1-hour breakout sessions included the following topics: introductory basic terminology and theory as they relate to transgender health (Transgender 101); disparities and ways to improve the trans experience in health care through gender-affirming care; addiction and the transgender population; implications for the pediatric population and family support; transitioning in the school setting; medical affirming hormones and gender confirmation surgery. All students then attended a panel discussion that included at least four trans community members eager to discuss their personal narratives and healthcare experiences in an open question-and-answer forum.

**Figure 1:**
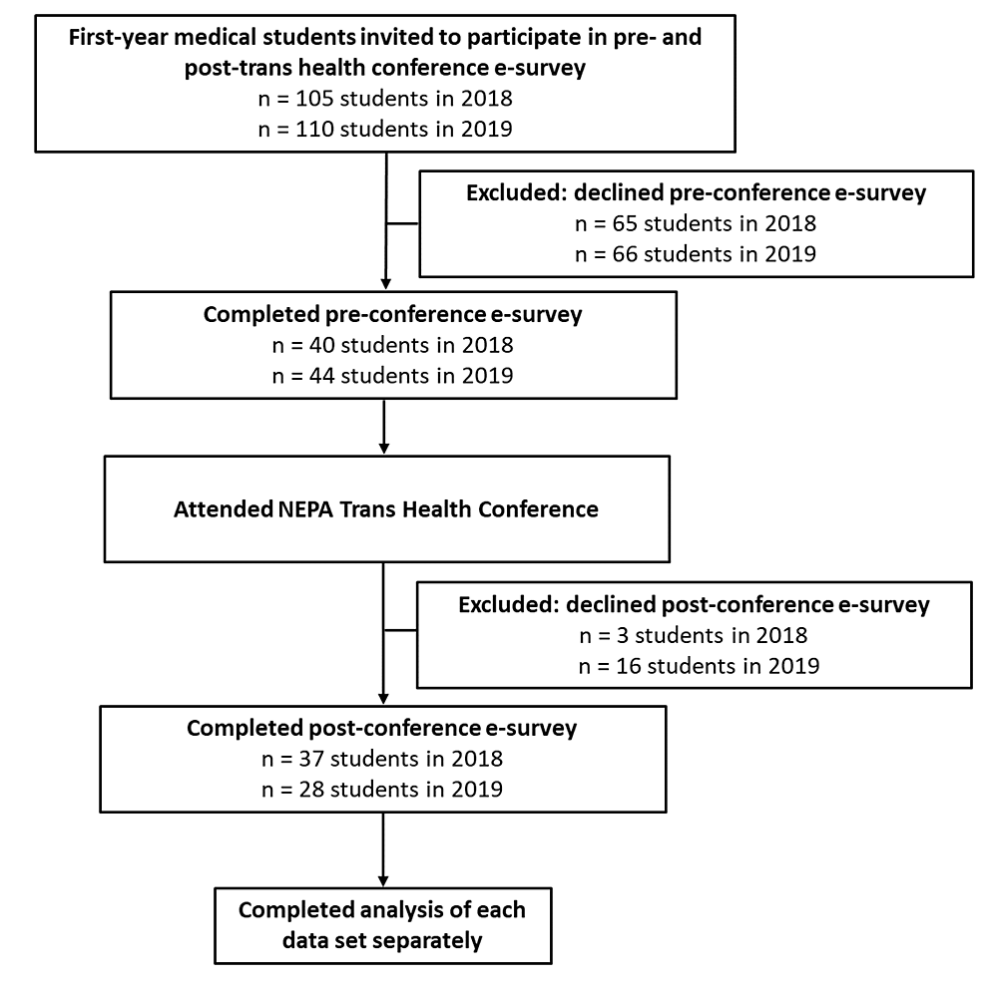
Flow chart of research design implemented for the 2018 and 2019 NEPA Trans Health Conferences.

Prior to the conference, students were informed about the study via verbal announcements in class. During this time, students were told of the purpose and participation instructions, including the anonymity and security of data collection. Students were instructed that although a direct identifier would be initially collected to match pre- and post-data by a data broker, this direct identifier would be removed by the data broker and not included in the final data set analyzed by study personnel. Students were also made aware that participation was voluntary and that decisions to participate had no repercussions on class standing. The research instrument consisted of a non-validated pre-and post-survey that was disseminated via email with an embedded link for survey participation and consent information. Pre-surveys were sent to all first-year students two weeks before the conference. Post-surveys were made available for students to answer by email, for one week following the conclusion of the conference. This outlined procedure was kept constant for both the 2018 (first year of the survey) and 2019 (second year of the survey) conferences; see Figure 1 below.

Survey 

The main purpose of the pre- and post-conference surveys was to help identify and understand possible changes in the knowledge, attitudes, and beliefs, of first-year medical students concerning the transgender community and their healthcare needs. Students were emailed a pre-survey before the conference and a post-survey at the conclusion of the conference. Due to the paucity of validated questionnaires specifically examining the attitudes, beliefs, and medical knowledge of medical students with respect to the healthcare concerns of trans people and patients in 2018, our group designed, and attempted to validate both sets of pre- and post-intervention surveys used in 2018 and 2019.

2018 survey

The survey used in the first year, Attitudes Toward Transgender Healthcare, was largely focused on student attitudes, beliefs, and perceptions (Appendix 1). This survey adapted questions from the Knowledge of Lesbians, Gays, Bisexuals, and Transgender People (KLGBT) questionnaire [27], the Attitudes Toward Lesbian, Gay, Bisexual and Transgender Patients (ATLGBTP) scale [27], as well as questions developed by our study team. The first-year survey focused on common misconceptions due to a lack of awareness or exposure to accepted LGBTQIA2S+ terminology, proper pronoun usage, the ability to discern gender from sexual orientation, and an incomplete understanding of the challenges faced by transgender people seeking competent and equitable health care. The survey included eight questions measured via a 5-point Likert-Scale (strongly agree = 5, agree = 4, undecided = 3, disagree = 2, and strongly disagree = 1). 

2019 survey

The survey used in the second year, Modified Attitudes Toward Transgender Healthcare, focused more on examining student attitudes regarding the provision of appropriate population-specific medical care as well as confidence levels in treating a transgender patient (Appendix 2). The instrument aimed to identify gaps in medically related knowledge, such as asking students to reflect on physician responsibility and perception, understanding basic principles of hormone therapy or sexual reassignment surgery, and knowledge of discrimination in the healthcare arena. Though drawing some inspiration from the KLGBT and ATLGBTP surveys [27], all questions were developed by our study team due to the lack of an established transgender healthcare-specific survey tool, at the time. The survey comprised of 21 questions, all of which were measured via a 5-point Likert Scale, (strongly agree = 5, agree = 4, undecided = 3, disagree = 2, and strongly disagree = 1).

It should be noted that although the first- and second-year surveys shared one similar question in addition to comparable subject matter, these tools were different in the overall outlook. As such, no comparisons or correlations were drawn between the 2018 and 2019 conference years. Self-reported student data collected from these surveys were used as tools for understanding whether the proposed intervention induced any changes in student attitudes, beliefs, or knowledge between pre- and post-survey. It should also be noted that internal analysis of our 2018 and 2019 survey questionnaires revealed a low average Cronbach’s alpha for both pre- and post-intervention surveys (average α = 0.59 for 2018 and average α < 0.5 for 2019).

Data collection and analysis

The pre-conference survey was sent out to all first-year medical school students two weeks before the conference and closed 24 hours prior to the start of the conference. The post-survey was sent to all students immediately following the conference and closed one week after the conclusion of the conference. Student feedback was embedded into the administered post-survey. Pre- and post-conference surveys were sent to all first-year students via Survey Monkey by a data broker who preserved the anonymity of participants by coding and matching individual pre- and post-conference participant responses. Although a direct identifier was collected to match pre- and post-data, this direct identifier was removed by the data broker and so not included in the final data set analyzed by study personnel. This timeline was maintained for both conference years. Likert-scale response data were transformed into numerical scores (strongly agree = 5, agree = 4, undecided = 3, disagree = 2, and strongly disagree = 1). Numerical scores from the pre- and post-surveys were then analyzed via paired t-tests. Effect size data was expressed in terms of Cohen's d, with d = 0.2, interpreted as small; d = 0.5, interpreted as medium, and d = 0.8, interpreted as large. The data presented in the results section were analyzed using GraphPad Prism version 9.5.1 for Windows (GraphPad Software, San Diego, California). Cronbach's alpha analysis was completed with Microsoft Excel.

## Results

Our results for both years demonstrate that the medical students largely held favorable attitudes towards caring for individuals who identify as transgender prior to attending each conference. For instance, students consistently reported feeling comfortable interacting with and providing health care to transgender patients both pre- and post-conference (Tables 1, 2). Students also responded positively and respectfully to the survey item suggesting preferred personal pronoun usage pre- and post-intervention (Table 2).

**Table 1:** 2018 Pre-/Post-Survey Matched Comparison of Medical Students Reporting Attitudes Towards Transgender Healthcare, N= 37. *t: *t*-score; **df: degrees of freedom

Survey Item	Pre-Survey Mean ± SD	Post-Survey Mean ± SD	t*, df**	p-value	Cohen’s d
I am knowledgeable about transgender health issues.	2.46 ± 0.869	3.27 ± 0.962	5.84, 36	<0.0001	0.88
I am sensitive to the fears of intolerance and discrimination transgender individuals feel when seeking healthcare.	4.11 ± 0.658	4.38 ± 0.594	2.71, 36	0.010	0.43
Being transgender is a natural expression of gender identity in men and women.	3.65 ± 0.919	3.93 ± 1.01	3.15, 36	0.003	0.29
A person who feels that their sex (male or female) does not match their gender identity (masculine or feminine) is just plain wrong.	1.49 ± 0.692	1.43 ± 0.765	0.442, 36	0.661	0.08
I feel prepared to treat a transgender person.	2.38 ± 0.893	2.95 ± 1.03	3.60, 36	0.0009	0.59
I would feel comfortable treating a transgender person.	4.14 ± 0.673	4.16 ± 0.646	0.329, 36	0.744	0.03
I understand the complexities of hormone therapy and sex reassignment surgery.	2.05 ± 0.848	3.16 ± 1.09	7.44, 36	<0.0001	1.14
I feel that my medical education is preparing me to understand the unique aspects related to caring for transgender individuals.	3.22 ± 0.821	3.35 ± 0.789	0.797, 36	0.431	0.16

**Table 2:** 2019 Pre-/Post-Survey Matched Comparison of Medical Students Reporting Modified Attitudes Towards Transgender Healthcare, N= 28. *t: *t*-score; **df: degrees of freedom

Survey Item	Pre-Survey Mean ± SD	Post-Survey Mean ± SD	t*, df**	p-value	Cohen’s d
I am knowledgeable about transgender healthcare needs.	3.19 ± 0.94	3.92 ± 0.74	4.05, 25	0.0004	0.86
I am sensitive to the fears of intolerance and discrimination transgender individuals feel when seeking healthcare.	4.26 ± 0.76	4.52 ± 0.51	2.05, 26	0.050	0.40
Gender identity and sexual orientation are two names for the same concept.	1.73 ± 0.96	1.31 ± 0.47	2.40, 25	0.0246	0.56
As an individual, I would feel comfortable interacting with an individual who identifies as transgender.	4.22 ± 0.93	4.18 ± 0.88	0.143, 26	0.887	0.04
An individual must be diagnosed with gender dysphoria by a mental health professional to receive hormone replacement therapy.	2.84 ± 0.88	2.77 ± 1.4	0.385, 25	0.7029	0.06
I understand the basic principles of hormone therapy and sex reassignment surgery.	3.00 ± 1.06	3.96 ± 0.72	4.03, 25	0.0005	1.06
I would feel comfortable providing health care for a transgender patient.	4.18 ± 0.91	4.07 ± 0.72	0.619, 27	0.541	0.14
An individual who identifies as transgender should be addressed using pronouns of their preferred gender rather than those referring to their natal sex.	4.38 ± 0.98	4.62 ± 0.70	1.44, 25	0.161	0.28
I know the difference between puberty blockers and cross hormone therapy and when it is appropriate to suggest or prescribe either.	1.89 ± 0.685	3.50 ± 1.20	6.47, 27	<0.0001	1.65
To be considered transgender, an individual must first undergo at least one sexual organ reassignment surgery.	1.65 ± 0.689	1.31 ± 0.55	2.37, 26	0.025	0.55
I feel prepared to take a medical history of a transgender patient.	3.32 ± 0.905	3.89 ± 0.83	3.29, 27	0.003	0.67
Transgender medicine is not an appropriate topic for conventional medicine at this time.	1.44 ± 0.698	2.15 ± 1.51	2,38, 26	0.025	0.60
I am knowledgeable about the specific health disparities and inequities that patients who identify as transgender face in our society.	3.63 ± 0.79	4.04 ± 0.59	3.05, 27	0.005	0.59
All physicians have a responsibility to treat patients who identify as transgender.	4.18 ± 1.19	4.25 ± 1.27	0.700, 27	0.489	0.06
I would feel comfortable if my professional peers knew I treated transgender patients.	4.48 ± 0.75	4.59 ± 0.69	1.00, 26	0.327	0.15
I am concerned that if my cis-gender patients learn I am treating transgender patients, they may no longer seek my care.	1.75 ± 0.80	1.68 ± 0.86	0.570, 27	0.573	0.08
I anticipate refusing to see patients for transgender health care based on the possible complexity involved.	1.71 ± 0.98	1.71 ± 0.98	0.00, 27	>0.999	0
I anticipate discomfort with providing male to female transgender (MTF) care.	2.04 ± 1.17	2.04 ± 1.16	0.00, 27	>0.999	0
I anticipate discomfort with providing female to male transgender (FTM) care.	2.04 ± 1.17	2.14 ± 1.27	0.500, 27	0.621	0.08
Transgender healthcare is an essential part of my medical training and belongs in the medical curriculum.	4.10 ± 0.88	4.10 ± 1.13	0.000, 27	>0.999	0
I feel that my medical education is preparing me to provide competent and compassionate care for patients that identify as transgender.	3.36 ± 1.03	3.89 ± 1.07	2.06, 27	0.049	0.51

2018 NEPA Trans Health Conference survey results

Of the 105 students who attended the conference, 60.0% (63/105) completed the pre-survey and 38.1% (40/105) of students responded to the post-survey. Of the conference attendees who completed the surveys, 35.24% (37/105) completed both the pre- and post-survey (matched students). When examining matched student data, 87.5% (7/8) of survey items demonstrated increases in the mean attitude as a measure of the Likert scale scores (Table 1). These items included attitudes about knowledge of transgender health issues, sensitivity to the fears of intolerance and discrimination patients face in seeking healthcare, understanding the transgender identity, feelings of preparedness and comfort in treating transgender patients, understanding the complexities of treatment plans, and the feeling that their medical education is preparing them to understand how to care for transgender individuals. In contrast, 12.5% (1/8) of survey items showed decreases in mean attitude (Table 1). This survey question asked students if a person who feels that their gender identity does not match their sex is “just plain wrong” (Appendix 1).

Overall, 62.5% (5/8) of survey items yielded statistically significant results (p-value ≤ 0.05) (Table 1). These survey items demonstrated that compared to before the conference, students now had increased knowledge about transgender health issues, increased sensitivity to the fears of intolerance and discrimination patients face in seeking healthcare, an increased understanding that being transgender is a natural expression of gender identity, increased preparedness in treating transgender patients, and increased understanding of the complexities of hormone therapy and sex reassignment surgery. Our results for both years demonstrate that the medical students largely held favorable attitudes towards caring for individuals who identify as transgender prior to attending each conference. For instance, students consistently reported feeling comfortable interacting with and providing health care to transgender patients both pre- and post-conference (Tables 1, 2). Students also responded positively and respectfully to the survey item suggesting preferred personal pronoun usage pre- and post-intervention (Table 2).

2019 NEPA Trans Health Conference survey results

Of the 110 students who attended the conference, 40.0% (44/110) completed the pre-survey and ­­­ 26.3% (29/110) of students responded to the post-survey. Of all the student-conference attendees, 25.5%, (28/110) completed both the pre- and post-survey (matched students). While examining matched student data, 57.1% (12/21) of survey items demonstrated increases in the mean attitude, again, as a measure of the Likert scale scores (Table 2). These survey items included attitudes regarding knowledge about transgender healthcare needs, increased sensitivity to the fears of intolerance and discrimination patients who identify as transgender, feel when seeking healthcare, understanding basic principles of hormone therapy and sex reassignment surgery, addressing individuals as their preferred gender pronouns, knowing the differences between and when to prescribe puberty blockers and cross hormone therapy, preparedness in taking a medical history of a transgender patient, the appropriateness of transgender medicine in conventional medicine at this time (in 2019), knowledge about health disparities and societal inequities, physician responsibility in treating transgender individuals, comfort with professional peers knowing the student will treat transgender patients, discomfort in providing female to male care, and feeling that their medical education is preparing them to provide competent and compassionate care for transgender patients. In contrast, 28.6% (6/21) of survey items showed decreases in mean attitude (Table 2). These survey items examined students’ attitudes and beliefs about recognizing the difference between the terms gender identity and sexual identity, interacting with transgender individuals, whether individuals must be diagnosed with gender dysphoria to receive hormone replacement therapy, their comfort level in providing healthcare for a transgender patient, the role surgery plays, if any, when identifying as transgender, and the possibility of cis-gender patients choosing not to seek medical care from a provider who treats transgender patients. In addition, 14.3% (3/21) of survey items demonstrated no changes in mean attitudes or beliefs (Table 2). These items indicated that students did not feel differently regarding the refusal to see patients seeking transgender healthcare due to the possible complexity and discomfort in providing male-to-female transgender care, and transgender healthcare belonging to the students’ medical curriculum.

Overall, 47.6 % (10/21) of survey items yielded significant results (Table 2). Evaluation of the pre- and post-survey data indicated that after attending the 2019 trans health conference, first-year medical ­students had increased knowledge about transgender healthcare needs, increased sensitivity to the fears of intolerance and discrimination patients face, decreased agreement that gender identity and sexual orientation are the same concepts, increased understanding of the basic principles of hormone therapy management and sex reassignment surgery, increased knowledge in knowing the differences between, and when to prescribe puberty blockers and cross hormone therapy; decreased agreement that an individual, must undergo sexual organ reassignment surgery in order to be considered transgender; increased preparedness in taking the history of a transgender patient, increased agreement that transgender medicine is not an appropriate topic for conventional medicine at this time, increased knowledge about health disparities and inequities, and increased agreement that their medical education is preparing them to provide competent and compassionate care for patients that identify as transgender.

## Discussion

This novel study uses two complementary datasets to provide evidence of statistically significant improvements associated with large effect sizes, in first-year medical student knowledge, attitudes, and beliefs regarding the transgender community and transgender healthcare concerns (p- and Cohen’s d values in Tables 1, 2). In both intervention years, the NEPA Trans Health Conference provided all subjects surveyed with an educational forum for healthcare professionals, their staffs, educators, and the general public to learn from the transgender community and from those who treat and support them about the necessary affirming care, respect, and understanding of people who are transgender. This study is also novel for its ability to investigate the effects of such a unique and effective informational gathering offering numerous opportunities for meaningful conversations between the transgender community and medical students in training. In addition to completing the pre- and post-survey in this study, students attended at least three one-hour educational sessions of the NEPA Trans Health Conference, ranging from introductory transgender terminology to training designed to improve the trans health care experience for pediatrics and adults alike, to topics like transitioning in the school setting, to informational sessions on medical affirming hormones and gender confirmation surgery. Students also attended a question-and-answer panel discussion conducted by four trans community members who appeared genuinely enthusiastic to share their personal narratives that often provided key information regarding their transgender healthcare journey. By attending these introductory, informational sessions coupled with asking questions and listening to patient narratives, students were offered insight into the fundamentals of approaching trans health patient care empathetically and competently through a population-specific approach. Population-specific exposures like this conference allow students to gain collaborative knowledge and insight directly from individual narratives and educational forums [28].

Our results for both years demonstrate that the medical students largely held favorable attitudes towards caring for individuals who identify as transgender prior to attending each conference. While it is very possible that medical students were hesitant to express negative attitudes about transgender people even in the presence of a data broker that eventually removed their direct identifier before any data was analyzed by research personnel, we are also of the opinion that these attitudes in part, speak to the innate compassionate, sensitive, respectful, and responsive character traits of first-year medical students dedicated to their institution’s curriculum that integrates and emphasizes social justice and health equity.

Additionally, we found that for both conference years, post-intervention data showed overall statistically significant changes in medical students’ attitudes about their soft skills regarding the transgender community (Tables 1, 2). Students demonstrated significant improvements in empathy and sensitivity toward a patient population that they previously had little exposure. Students in both surveys reported having greater sensitivity to the fears of intolerance and discrimination transgender individuals feel when seeking healthcare, after attending the trans health conferences. Thus, conferences such as these have the capacity to reinforce and value the importance of personal experiences, formative histories, and cultural norms that intersect with LGBTQIA2S+ culture and individuals’ identities [2,29].

Though the 2018 and 2019 surveys did not differ in terms of thematic outlook, the 2019 survey was more expansive in identifying nuances within student attitudes. For example, following the 2019 conference, students reported feeling less comfortable providing healthcare for a transgender patient, while also disagreeing ‘less intensely’ that transgender medicine was not an appropriate topic at this time. Yet, the same 2019 survey analysis demonstrated that attendance at the NEPA Trans Health Conference, yielded significantly positive results in terms of improving student knowledge, as students reported being more knowledgeable about transgender healthcare needs, as well as being able to discern between the terms gender identity and sexual orientation. Students also reported having a greater understanding of the basic principles of hormone therapy and sex reassignment (gender affirmation) surgery (Table 2).

Based on these and additional observations, in which both the 2018 (5/8), and 2019 (9/21) survey items yielded pre-and post-conference significant differences in responding, we believe interventions, such as the NEPA Trans Health Conference, provide a compelling space in which the transgender community can direct and participate in an educational conversation about their healthcare journeys with medical students who will ultimately be directly involved in their care, and vice versa. However, it will be important in future studies to further probe these assertions by providing in tandem, a comprehensive analysis of conference-attendee evaluations in addition to our pre-and post-intervention findings. Nonetheless, at this time, we confidently support the notion that as medical schools plan and work to improve curricula, they should consider the prevalence of LGBTQIA2S+ patients and professionals in their own communities and follow suit by providing similar interventions that utilize the expertise of these individuals and communities to educate their students. Integrating and introducing such interventions in the medical school curriculum, especially relevant exposures that are specifically tailored to the level of training the student is at, can allow medical students to develop into competent and compassionate clinical providers [17].

This study offers support towards integrating a community-based approach to exposing medical students to human complexity and diversity that basic sciences and clinical curricula are challenged to present holistically. To this day, the lack of knowledgeable providers is the leading barrier to providing compassionate and quality care to transgender patients [16]. Despite a movement to improve health care quality, clinicians continue to hold strong biases that are perpetuated by stereotypes, assumptions, and frankly misinformation, regarding the transgender narrative [30,31]. Clinicians who are poorly educated or biased about gender diversity may be deliberately or accidentally discriminatory in their words or behavior [32]. Such clinicians can add to and perpetuate institutional and systemic discrimination leading to the denial of the existence of transgender and gender-diverse individuals, with consequential negative impacts on their health outcomes and access to competent and compassionate healthcare [32]. Education and structured training interventions remain the most fundamental method in closing the barrier that exists in providing sustainable increases in knowledge and improvements in compassionate patient-forward care towards the transgender patient populations [33-36]. Incorporating trans-health into the medical school curriculum, such as focused history-taking, clerkships, and endocrinology and psychiatry lectures, is strongly endorsed by the World Health Organization (WHO) and the AAMC (Association of American Medical Colleges) [34,35]. Medical school curricula, health team education, and research initiatives that focus on cultural humility, engagement in community leadership, holistic longitudinal health outcomes, and practice-focused training are key elements towards achieving equitable affirmative care for all [32,37-40].

This original study is limited by non-representative samples of primarily first-year medical students (N = 28-37/110 students/conference year), who volunteered to participate from a single institution. In addition to the voluntary nature of the survey, first-year-student-focus on basic science knowledge and preparation for both in-house and national board exams (2018 and 2019, prior to the Step 1 transition to pass-fail), may have contributed to the reduced number of post-survey respondents. The inclusion of clinical students should be considered in future studies, as their training, acquired knowledge, and clinical experience may result in more meaningful, seasoned responses and, therefore, more relevant and concrete data to consider.

This study is also limited for not collecting and including essential demographic information like age, race, ethnicity, culture, gender, marital status, sexual orientation, having LGBTQIA2S+ contacts, religion, and previous transgender health-related education of the first-year medical students surveyed. Exclusion of such information can be problematic because it can infer a posture of presumed absolutism that discounts the significant influence that any of the above-mentioned demographic pieces of information can have on subject responses and, thus, conclusions [41,42]. Without the concomitant scrutiny of demographic variables in studies like this, the reader risks being misinformed.

In addition to our data being somewhat dated from 2018 and 2019, internal analysis of our survey questionnaires revealed a low average Cronbach’s alpha for both pre- and post-intervention surveys (average α = 0.59 for 2018 and average α < 0.5 for 2019), thus casting a greater degree of suspicion over the reliability and thus the validity of our questionnaire, prompting us to again rethink and retool our survey questions in future studies.

Despite these limitations, however, this study provides insight into how medical institutions may better train future clinicians in order to competently treat symptoms and reduce any stigma surrounding transgender patients. The care of gender-diverse and transgender people has unfortunately become a culture war topic in the US and contentious internationally [43,44]. It is crucial, now more than ever, that the education of a future generation of physicians optimally prepares them to provide the best possible gender-affirming and compassionate care for gender-nonconforming people by partnering with local LGBTQIA2S+ leadership to design programs like the NEPA Trans Health Conference when their core curriculum lacks specific exposure.

## Conclusions

By providing a yearly trans health medical conference along with pre-post assessments within a medical school curriculum, this report found significant improvements in first-year medical student attitudes and knowledge about the noteworthy healthcare needs of gender-diverse and transgender patients. Delivering a narrative-forward humanistic outlook within the medical curriculum offers a bridge between academic content and providing altruistic healthcare. Furthermore, when physician providers have the gender-affirming tools to effectively offer their transgender patients’ affirmation, acceptance, and medical guidance, patients feel supported during their healthcare journey.

## Appendices

**Table 3:** 2018 Survey: Attitudes Toward Transgender Healthcare

Survey Item	Strongly Disagree 1	Disagree 2	Undecided 3	Agree 4	Strongly Agree 5
I am knowledgeable about transgender health issues.					
I am sensitive to the fears of intolerance and discrimination transgender individuals feel when seeking healthcare.					
Being transgender is a natural expression of gender identity in men and women.					
A person who feels that their sex (male or female) does not match their gender identity (masculine or feminine) is just plain wrong.					
I feel prepared to treat a transgender person.					
I would feel comfortable treating a transgender person.					
I understand the complexities of hormone therapy and sex reassignment surgery.					
I feel that my medical education is preparing me to understand the unique aspects related to caring for transgender individuals.					

**Table 4:** 2019 Survey: Modified Attitudes Toward Transgender Healthcare

Survey Item	Strongly Disagree 1	Disagree 2	Undecided 3	Agree 4	Strongly Agree 5
I am knowledgeable about transgender healthcare needs.					
I am sensitive to the fears of intolerance and discrimination transgender individuals feel when seeking healthcare.					
Gender identity and sexual orientation are two names for the same concept.					
As an individual, I would feel comfortable interacting with an individual who identifies as transgender.					
An individual must be diagnosed with gender dysphoria by a mental health professional to receive hormone replacement therapy.					
I understand the basic principles of hormone therapy and sex reassignment surgery.					
I would feel comfortable providing health care for a transgender patient.					
An individual who identifies as transgender should be addressed using pronouns of their preferred gender rather than those referring to their natal sex.					
I know the difference between puberty blockers and cross hormone therapy and when it is appropriate to suggest or prescribe either.					
To be considered transgender, an individual must first undergo at least one sexual organ reassignment surgery.					
I feel prepared to take a medical history of a transgender patient.					
Transgender medicine is not an appropriate topic for conventional medicine at this time.					
I am knowledgeable about the specific health disparities and inequities that patients who identify as transgender face in our society.					
All physicians have a responsibility to treat patients who identify as transgender.					
I would feel comfortable if my professional peers knew I treated transgender patients.					
I am concerned that if my cis-gender patients learn I am treating transgender patients, they may no longer seek my care.					
I anticipate refusing to see patients for transgender health care based on the possible complexity involved.					
I anticipate discomfort with providing male to female transgender (MTF) care.					
I anticipate discomfort with providing female to male transgender (FTM) care.					
Transgender healthcare is an essential part of my medical training and belongs in the medical curriculum					
I feel that my medical education is preparing me to provide competent and compassionate care for patients that identify as transgender.					
